# Increased risk of tinnitus in patients with early-onset cataracts: a nationwide population-based case-control study

**DOI:** 10.1186/s12886-020-01497-8

**Published:** 2020-06-16

**Authors:** Yi-Ching Hsieh, Cheng-Hsien Chang, Yi-Yu Tsai, Cheng-Li Lin, Telk-Ying Ng, Chun-Chi Chiang

**Affiliations:** 1 Department of Ophthalmology, China Medical University Hospital, China Medical University, No. 2, Yude Road, Taichung, Taiwan; 2 School of Medicine, China Medial University, Taichung, Taiwan; 3 Management Office for Health Data, China Medical University Hospital, Taichung, Taiwan; 4 Department of Public Health, China Medical University College of Public Health, Taichung, Taiwan; 5 Department of Otolarygology, Head and Neck Surgery, China Medical University Hospital, China Medical University, Taichung, Taiwan

**Keywords:** Early-onset cataract, Tinnitus, Cohort study, Case-control, Risk

## Abstract

**Background:**

This study aimed to investigate the association between early-onset cataract and tinnitus using a population-based database.

**Methods:**

Retrospective claims data from the Taiwan National Health Insurance Research Database were analysed. Study subjects comprised patients with early-onset cataract, aged 20–55 years and diagnosed between 2000 and 2010 (*n* = 2084) and a comparison cohort without the disease (*n* = 8336). Both cohorts were followed until 2010 to estimate the incidence of tinnitus. To calculate the risk of tinnitus in the case and control groups, Cox proportional hazards models were used and presented as hazard ratios (HRs), adjusted HRs (aHRs) and 95% confidence intervals (CIs).

**Results:**

Patients with early-onset cataract had 1.53-fold increased risk (HR = 1.53, 95% CI = 1.17–2.01, *p* < 0.01) of developing tinnitus than controls. The number of patients with vertigo (*p* < 0.0001), insomnia (*p* < 0.0001), anxiety (*p* < 0.0001) and hearing loss (*p* < 0.0001) as comorbidities was also significantly higher in the case group. After adjusting for age, sex and all listed comorbidities, patients with increasing age (aHR = 1.04, 95% CI = 1.02–1.07), early-onset cataract (aHR = 1.32, 95% CI = 1.01–1.74), vertigo (aHR = 1.75, 95% CI = 1.15–2.67), insomnia (aHR = 1.48, 95% CI = 1.14–1.93) and hearing loss (aHR = 6.20, 95% CI = 3.58–10.70) had significantly higher risk of tinnitus.

**Conclusions:**

Patients with early-onset cataract are at an increased risk of developing tinnitus in subsequent years and should receive further evaluation for early diagnosis and management if any signs of tinnitus occur.

## Background

Cataract is defined as the opacity of the eye lens. It is also the leading cause of blindness, accounting for 51% of cases of blindness globally according to the latest assessment (2010) [1]. The opacity of the lens directly results from oxidative stress [2]. Oxidative stress is an imbalance between the rate of oxidant production and the rate of its degradation. The loss of glutathione (GSH), the primary and essential lenticular antioxidant, is a crucial feature in age-related nuclear cataract [3]. Age-related cataracts usually develop in patients aged > 60 years; however, cataracts may also develop in some patients aged < 55 years [4]. Multiple risk factors are responsible for the development of early-onset cataract, such as diabetes, high myopia, steroid use, smoking and ultraviolet (UV) light exposure [5–9]. Oxidative stress may be one of the major contributing factors in early-onset cataract. Other pathogeneses of early-onset cataract include congenital, idiopathic, chronic inflammation, traumatic and cataract related to systemic diseases.

Tinnitus, defined as the subjective perception of noises in the absence of an external auditory stimulus, is a multifactorial disorder. The reported prevalence of tinnitus in the general population is approximately 10 to 15% [10]. There are several causes of tinnitus, including otologic, neurologic and infectious causes [11, 12]. Hearing loss has been considered the most common cause [13]. Moreover, several authors have observed that reactive oxygen species (ROS) plays a role in the pathogenesis of tinnitus [14–16].

Because oxidative stress is related to both early-onset cataract and tinnitus, the present study investigates whether patients with early-onset cataracts are at risk of tinnitus using the nationwide population-based dataset from Taiwan.

## Methods

### Data source

Taiwan established a nationwide database, the National Health Insurance Research Database (NHIRD), in 1995. Presently, this database contained comprehensive medical information of > 99% of single-payer insurance beneficiaries in Taiwan. The medical information included the historical record of out- and inpatients and medications but without identification to protect the privacy of each subject.

In this study, we used the Longitudinal Health Insurance Database, which selected one million subjects from the NHIRD by randomization.

All previous diagnoses in the database were coded according to the *International Classification of Disease*, *Ninth Revision, Clinical Modification* (ICD-9-CM). The Research Ethics Committee of China Medical University and Hospital in Taiwan approved the study protocol (CMUH-104-REC2–115-R3).

### Study population

Based on the study objective, we aimed to confirm the association between early-onset cataract and tinnitus. We selected patients with early-onset cataract (ICD-9-CM: 366.00, 366.01, 366.02, 366.03, 366.04, 366.09, 366.17 and 366.18) as the case group and defined the diagnosis date as the index year; the control group was selected by 4:1 matching with the case group, and the matching variables included age, sex and index year. The enrollment date for the subject in the control group was matched with the same year of the subject in the early-onset cataract cohort, while the month and the day was randomly assigned. The study population was followed until the development of tinnitus, lost to follow-up or 31 December 2011 and the study period was from 2000 to 2011. A total of 2193 patients aged 20–55 years with newly-diagnosed early-onset cataract were identified in our study. Patients those with a history of tinnitus (ICD-9-CM: 388.3) (*n* = 109) before the index date were excluded from our study.

The comorbidities evaluated in this study were vertigo (ICD-9-CM: 386), insomnia (ICD-9-CM: 780), anxiety (ICD-9-CM: 300.00) and hearing loss (ICD-9-CM: 388–389).

### Statistical analysis

This study included demographic, residential and comorbidities variables. The continuous and categorical variables were presented as mean ± standard deviation and number (%), respectively. To compare the difference of each variable in the two groups, t-test and chi-square test were used, respectively. To calculate the risk of tinnitus in the case and control groups, Cox proportional hazards models were used and presented as hazard ratios (HRs), adjusted HRs (aHRs) and 95% confidence intervals (CIs). The cumulative incidence of tinnitus in the two groups was obtained using the Kaplan–Meier method, and the log-rank test was used to compare the cumulative incidences in the two groups. All statistical analyses were performed using SAS version 9.4 (SAS Institute Inc., Cary, NC). The cumulative incidence curve was plotted using R software. The significance level was set at a two-sided *P*-value < 0.05.

## Results

There were a total of 10,420 eligible participants in this study: 2084 patients with early-onset cataract (case) and 8336 individuals without early-onset cataract (control) (Table 1). In this study, 50.1% of the study subjects were men, and the mean age in the case and control groups was 46.3 and 45.4 years, respectively; most of them were aged between 46 and 55 years (67.0%) and living in the highest urbanization level region. There were no significant differences in sex, age and index year between the two groups. The number of patients with vertigo (*p* < 0.0001), insomnia (p < 0.0001), anxiety (p < 0.0001) and hearing loss (p < 0.0001) as comorbidities was significantly higher in the case group. The mean follow up years of the early-onset cataract cohort is 4.76 (±SD 3.12) years and of the non-early-onset cataract cohort is 4.82 (± SD 3.17) years.

**Table 1:** Comparisons in demographic characteristics and comorbidities in patient with and without Early-onset cataract

	Early-onset cataract		*p*-value
	No	Yes	
	(*N* = 8336)	(*N* = 2084)	
Gender			0.99
Women	4160 (49.9)	1040 (49.9)	
Men	4176 (50.1)	1044 (50.1)	
Age stratified			0.99
≤ 35	772 (9.26)	193 (9.26)	
36–45	1980 (23.8)	495 (23.8)	
46–55	5584 (67.0)	1396 (67.0)	
Age, mean ± SD ^a^	45.4 (7.43)	46.3 (7.41)	< 0.0001
Urbanization level^†^			0.02
1 (highest)	2601 (31.2)	670 (32.2)	
2	2558 (30.7)	682 (32.7)	
3	1558 (18.7)	329 (15.8)	
4 (lowest)	1619 (19.4)	403 (19.3)	
Comorbidity			
Vertigo	358 (4.29)	132 (6.33)	< 0.0001
Insomnia	2689 (32.3)	961 (46.1)	< 0.0001
Anxiety	589 (7.07)	222 (10.7)	< 0.0001
Hearing loss	45 (0.54)	28 (1.34)	< 0.0001

Chi-Square Test, ^a^ t-test

^†^The urbanization level was categorized by the population density of the residential area into 4 levels, with level 1 as the most urbanized and level 4 as the least urbanized

**Fig. 1:**
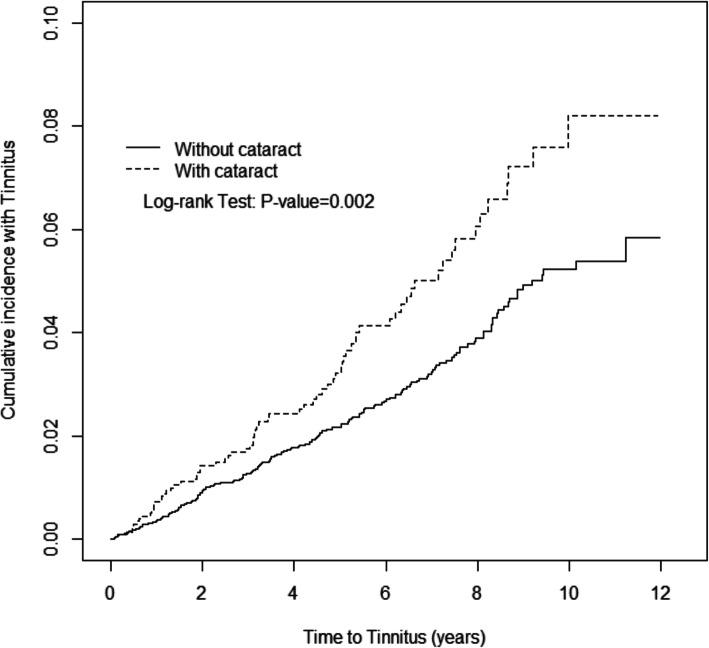
Cumulative incidence of Tinnitus for patients with (dashed line) or without (solid line) Early-onset cataract

Table 2 presents the incidence of tinnitus and HR between the case and control groups after stratification by demographic characteristics and comorbidities. The incidence rate of tinnitus in population without early-onset cataract was 4.83 (per 1000 person-years), and 7.36 in population with early-onset cataract. Patients with early-onset cataract had 1.53-fold increased risk (HR = 1.53, 95% CI = 1.17–2.01) of developing tinnitus than controls. Considering sex, stratified age, comorbidities and follow-up duration, patients with early-onset cataract who were women (HR = 1.43, 95% CI = 1.00–2.05), men (HR = 1.68, 95% CI = 1.11–2.53), aged < 35 years (HR = 7.97, 95% CI = 2.40–26.5) and aged between 36 and 45 years (HR = 2.50, 95% CI = 1.34–4.66), had no comorbidities (HR = 1.63, 95% CI = 1.09–2.44) and had a follow-up duration of > 4 years (HR = 1.70, 95% CI = 1.13–2.54) had significantly higher risk of developing tinnitus than controls. After adjusting for age, sex and comorbidities of vertigo, insomnia, anxiety and hearing loss, patients with early-onset cataract (aHR = 1.32, 95% CI = 1.00–1.73), aged < 35 years (aHR = 6.04, 95% CI = 1.74–21.0), aged between 36 and 45 years (aHR = 2.46, 95% CI = 1.31–4.61), without comorbidities (aHR = 1.58, 95% CI = 1.05–2.37) and followed for > 4 years (aHR = 1.62, 95% CI = 1.08–2.44) had higher risk of developing tinnitus than controls. Figure 1 shows that the cumulative incidence of tinnitus was significantly higher in patients with early-onset cataract than in controls (*p* = 0.002).

**Table 2:** Comparison of incidence densities of Tinnitus and hazard ratio between with and without Early-onset cataract by demographic characteristics and comorbidity

	Early-onset cataract							
	No			Yes				
	Event	PY	Rate^#^	Event	PY	Rate^#^	Crude HR*(95% CI)	Adjusted HR^†^ (95% CI)
All	194	40,186	4.83	73	9913	7.36	1.53 (1.17, 2.01)**	1.32 (1.00, 1.73)*
Gender								
Women	116	20,373	5.69	41	5043	8.13	1.43 (1.00, 2.05)*	1.25 (0.87, 1.79)
Men	78	19,813	3.94	32	4869	6.57	1.68 (1.11, 2.53)*	1.41 (0.93, 2.15)
Stratify age								
≤ 35	4	3872	1.03	8	966	8.28	7.97 (2.40, 26.5)***	6.04 (1.74, 21.0)**
36–45	26	9493	2.74	16	2341	6.84	2.50 (1.34, 4.66)**	2.46 (1.31, 4.61)**
46–55	164	26,821	6.11	49	6606	7.42	1.22 (0.89, 1.68)	1.09 (0.79, 1.50)
Comorbidity^‡^								
No	96	27,707	3.46	31	5476	5.66	1.63 (1.09, 2.44)*	1.58 (1.05, 2.37)*
Yes	98	12,479	7.85	42	4436	9.47	1.21 (0.84, 1.73)	1.20 (0.84, 1.73)
Follow-up time^‡^								
≤ 4 years	113	25,582	4.42	40	6404	6.25	1.42 (0.99, 2.03)	1.13 (0.78, 1.63)
> 4 years	81	14,604	5.55	33	3509	9.40	1.70 (1.13, 2.54)*	1.62 (1.08, 2.44)*

Rate^#^, incidence rate, per 1000 person-years; Crude HR*, relative hazard ratio; Adjusted HR^†^: multivariable analysis including age, gender, and comorbidities of vertigo, insomnia, anxiety, and hearing loss; *p < 0.05, **p < 0.01, ***p < 0.001

Comorbidity^‡^: Patients with any one of the comorbidities vertigo, insomnia, anxiety, and hearing loss were classified as the comorbidity group

^‡^The follow-up time is partitioned into 2 segments (years ≤4, and > 4 years) by median

Table 3 presents the risk factors of tinnitus. Study subjects who were women (HR = 1.39, 95% CI = 1.09–1.77) and had increasing age (HR = 1.05, 95% CI = 1.03–1.08), early-onset cataract (HR = 1.53, 95% CI = 1.17–2.01), vertigo (HR =2.62, 95% CI = 1.75–3.93), insomnia (HR = 1.94, 95% CI =1.52–2.47), anxiety (HR = 1.77, 95% CI = 1.19–2.63) and hearing loss (HR = 8.75, 95% CI = 5.11–15.0) had significantly higher risk of developing tinnitus. After adjusting for age, sex and all listed comorbidities, patients with increasing age (aHR = 1.04, 95% CI = 1.02–1.07), early-onset cataract (aHR = 1.32, 95% CI = 1.01–1.74), vertigo (aHR = 1.75, 95% CI = 1.15–2.67), insomnia (aHR = 1.48, 95% CI = 1.14–1.93) and hearing loss (aHR = 6.20, 95% CI = 3.58–10.70) still had significantly higher risk of developing tinnitus.

**Table 3:** Cox model with hazard ratios and 95% confidence intervals of Tinnitus associated with Early-onset cataract and covariates

	Crude		Adjusted^†^	
Variable	HR	(95% CI)	HR	(95% CI)
Gender				
Women	1.39	(1.09, 1.77)**	1.22	(0.95, 1.57)
Men	1	(reference)	1	(reference)
Age, years	1.05	(1.03, 1.08)***	1.04	(1.02, 1.07)***
Urbanization level				
1 (highest)	1.02	(0.71, 1.47)	–	–
2	1.27	(0.90, 1.81)	–	–
3	1.09	(0.73, 1.64)	–	–
4 (lowest)	1	(reference)	–	–
Baseline comorbidities (yes vs no)				
Early-onset cataract	1.53	(1.17, 2.01)**	1.32	(1.01, 1.74)*
Vertigo	2.62	(1.75, 3.93)***	1.75	(1.15, 2.67)**
Insomnia	1.94	(1.52, 2.47)***	1.48	(1.14, 1.93)**
Anxiety	1.77	(1.19, 2.63)**	1.22	(0.80, 1.84)
Hearing loss	8.75	(5.11, 15.0)***	6.20	(3.58, 10.7)***

Crude HR*, relative hazard ratio; Adjusted HR^†^: multivariable analysis including age, gender, and comorbidities of vertigo, insomnia, anxiety, and hearing loss

*p < 0.05, **p < 0.01, ***p < 0.001

## Discussion

The population aged > 55 years is more susceptible to lens opacity [17], and the prevalence of tinnitus is also increasing with age [18]. However, no previous study has demonstrated the relationship between early-onset cataract and the risk of tinnitus. The present retrospective cohort study found that the incidence rate of tinnitus in population without early-onset cataract was 4.83 (per 1000 person-years), and 7.36 in population with early-onset cataract. Moreover, the risk of tinnitus was 1.53-fold higher in patients with early-onset cataract than controls. In the category less than 4 years of follow-up, the incidence of tinnitus and hazard ratio between population with and without early-onset cataract demonstrated no statistical difference. The follow-up duration may not be long enough to detect whether early-onset cataract is a risk factor for tinnitus. In the other category with no statistical difference, age 46–55, the reason may be related to the age distribution of cataract and tinnitus. The prevalence of cataract increased with age and most occurred after age of 50 years, as well as tinnitus. As such, early-onset cataract may not be shown as a significant risk factor for tinnitus in the age group older than 50 years.

The number of patients with vertigo (*p* < 0.0001), insomnia (*p* < 0.0001), anxiety (*p* < 0.0001) and hearing loss (*p* < 0.0001) as comorbidities was significantly higher in patients with early-onset cataract than in controls. Insomnia, hearing loss and anxiety are common symptoms that are associated with tinnitus, and these symptoms are highly relevant to tinnitus severity [12, 19, 20]. In contrast, cataracts is not well-known associated with insomnia, anxiety and hearing loss.. Therefore, we select these associating factors to evaluate further correlation between early-onset cataract and tinnitus. In addition to our findings, there were few reports detecting the correlation between insomnia, anxiety, hearing loss and cataract respectively. The rate of anxiety of cataract patients is 18.0% in a Chinese-based study, which is in keep with cataract patients in Russia (20%), higher than the rate (7.0%) in healthy people reportedly [21, 22]. The BEAVER DAM study revealed that the combinations of cataract and hearing loss were common, occurring in 28% of the population 48–92 years, increasing consistently with age. In younger population (48–59 years), the prevalence of simultaneous cataract and hearing loss was 3.1%. Although risk is strongly related to age, some elective exposures appear to influence risk, such as heavy drinking and smoking [23]. The association between sleep and cataract is based on circadian rhythms. Cataract is opacity of the lens characterized by light scattering and absorption, especially the blue light. Thus, cataract may result in a decreased potential for circadian photoentrainment via retinohypothalamic tract, which also regulates the sleeping pattern. Decreased transmission of blue light to the retina in the opacified lens is significantly associated with an increased risk of sleep disturbances [24]. Alexander I et al. found that overall sleep quality and sleep latency of the patients improves after removal of cataract irrespective of the type of IOL implanted [25].

To further investigate the relationship between early-onset cataract and comorbidities of tinnitus, we analysed the risk factors of tinnitus based on the data in this large population-based study. After adjusting for possible confounding factors, patients with early-onset cataract still had significant higher risk of tinnitus, similar to those with increasing age, vertigo, insomnia and hearing loss.

Several possible correlations exist between early-onset cataract and tinnitus. Oxidative stress associated with UV light exposure plays an important role in the pathogenesis of cataracts. The loss of GSH, the essential and primary lenticular antioxidant, from the nuclear region is probably the crucial feature that precedes cataract formation [26]. Depletion of GSH allows low levels of oxidant to damage epithelial Na/K-ATPase and proteins associated with cell membrane permeability. Additionally, Boscia et al. [27] found that the ROS might initiate a cascade of toxic biochemical reactions such as peroxidation of the membrane lipids, which causes intracellular protein aggregation and precipitation, resulting in lens opacification. In tinnitus, high concentrations of ROS can have cytotoxic and neurotoxic effects, causing damage to the auditory hair cells of the labyrinth and acoustic system [28]. Neri et al. [29] reported that the levels of oxidative damage markers (malondialdehyde, 4-hydroxynonenal, myeloperoxidase) were higher in patients with tinnitus than in controls, whereas GSH peroxidase, an antioxidant enzyme, had lower activity in patients with tinnitus, sharing a similar pathogenesis with cataract.

Metabolic disorder, such as diabetes mellitus (DM), is another risk factor of early-onset cataract. In patients with DM, glucosylation of lens crystallins contributes to the accumulation of glycation end products, causing aggregation of high-molecular-weight material and resulting in lens opacification [30]. Studies have demonstrated that hyperglycaemia is associated with the impairment of cochlear microcirculation, especially in the stria vascularis. Ischaemia and hypoxia result in loss of outer hair cells and damage of neural units, leading to inner ear disorders, including tinnitus [31, 32]. In addition to DM, smoking is also a risk factor of both early-onset cataract and tinnitus [18, 33, 34]. Similar to the mechanism in DM, smoking-mediated vascular dysfunction and arteriosclerosis contribute to ischaemia and hypoxia in cochlear microcirculation [35]. Moreover, hydrogen cyanide, lead, styrene and toluene in cigarette are ototoxic agents [36]. Cyanide is also responsible for the development of early-onset cataract [37]. The effect of cyanide toxicity on Ca^2+^-ATPase activities may result in the disruption of vitreous and lenticular calcium homeostasis, causing Ca^2+^ accumulation and lenticular opacification [38].

Occupational exposure, including ionizing radiation exposure (radiologic technologists) and solar radiation exposure (outdoor worker), is a risk factor for cataract [39, 40]. In addition, excessive fuel exposure (solid cooking fuel combustion and poor kitchen ventilation) and metallic exposure (heavy metals work) are other possible risk factors associated with occupation [34, 41]. There are few epidemiological studies estimating occupational risk for tinnitus. Prevalence for working in noisy environment for > 10 years was estimated higher in both men and women in comparison with those with no occupational exposure to noise [42]. Engdahl B et al. found that occupation had significant effects on the prevalence of tinnitus [43]. Crane and hoist operators and miners in men, and laboratory assistants in women had the highest tinnitus prevalence. In women, occupations with the highest risks were not typically noisy ones, and housekeeper and the group of occupationally inactive also had higher prevalence. There might be an association between fuel exposure and tinnitus, like in cataract. No definite common occupational exposure exists in early-onset cataract and tinnitus in literature. Further research providing a better evaluation of occupation as risk factor is needed.

Steroids use is one of the cause of early-onset cataract. Nevertheless, no studies advocate long-term use of steroids in current management of tinnitus. There are no licensed drugs in Europe or North America for treatment of spontaneous idiopathic tinnitus [20]. Drugs from broad categories have been tested for effect on tinnitus; however, there is no standard treatments. For tinnitus with idiopathic sudden sensorineural hearing loss, intratympanic dexamethasone treatment has been showed effective in short term [44]. The other study demonstrated intratympanic drug therapy with steroid has resulted in both short- and long-term tinnitus relief in 7 of 10 patients (70%) identified to have a predominantly cochlear-type tinnitus [45]. However, there was no reports about benefit of long-term use of steroids for tinnitus. As such, steroids use may not be responsible for the correlation between early-onset cataract and tinnitus.

There are few limitations in this study. First, the diagnoses of early-onset cataract, tinnitus and comorbidities are completely dependent on ICD codes. However, the reliability is strengthened by the NHI Bureau of Taiwan. Hospitals receive heavy penalties from the NHI Bureau when overcharging, discrepancies and malpractice are detected. Second, this study is an observational retrospective cohort study, so a small number of patients may have undetected early-onset cataract or tinnitus and might have had a possibility of being categorised in the wrong cohort consequently. Nevertheless, the sample sizes are large in both the case and control groups, reducing the selection bias. Additionally, according to Taiwan National Health Insurance Annual Statistical database, 2010 annual contact rate of outpatient visits (per 100,000 population) under icd-9 code 360–379 based on age distribution were as follows: 32859 (0–4 years), 56,810 (5–9 years), 44,784 (10–14 years), 25,525 (15–19 years), 21,363 (20–24 years), 20,314 (25–29 years), 19,316 (30–34 years), 19,504 (35–39 years),20,770 (40–44 years), 21,695 (45–49 years) and 24,110 (50–54 years) [46]. According to a study, the medical facilities have good accessibility which Taiwanese citizens can see any doctor without a referral [47]. Also, the medical cost is quite low which seeing a doctor is not a huge burden for most citizens. Hence, people in Taiwan tend to seek a professional help when they have any tiny symptoms. Moreover, the data did not include contact rate of inpatient, and icd-9 code 360–379 did not represent all eye diseases. Thus, the true percentage of patients examined by an ophthalmologist is higher than the above data, and the rate of cataracts under-diagnosed is relative low.

## Conclusions

Patients with early-onset cataract have higher risk of developing tinnitus, similar to those of other well-known risk factors in this large population-based study. Of importance, patients should receive further evaluation for early diagnosis and management if any signs of tinnitus occur. Oxidative stress may be one of the main pathogeneses shared by early-onset cataract and tinnitus. Further studies should be conducted to verify whether the relationship is observed only in Asia or worldwide and to confirm the mechanisms involved.

## Data Availability

The datasets generated and analysed during the current study are from the National Health Insurance Research Database (NHIRD), which has been transferred to the Health and Welfare Data Science Center (HWDC). Interested researchers can obtain the data through formal application to the HWDC, Department of Statistics, Ministry of Health and Welfare, Taiwan (http://www.nhi.gov.tw/english/index.aspx).

## Abbreviations

HRs: Hazard ratios
aHRs): Adjusted HRs
CIs: Confidence intervals
GSH: Glutathione
UV: Ultraviolet
ROS: Reactive oxygen species
NHIRD: National Health Insurance Research Database
ICD-9-CM: International Classification of Disease, Ninth Revision, Clinical Modification
DM: Diabetes mellitus
